# Influence of Cucurbiturils on the Production of Reactive Oxygen Species by T- and B-Lymphocytes, Platelets and Red Blood Cells

**DOI:** 10.3390/ijms24021441

**Published:** 2023-01-11

**Authors:** Alina A. Aktanova, Olga S. Boeva, Margarita Sh. Barkovskaya, Ekaterina A. Kovalenko, Ekaterina A. Pashkina

**Affiliations:** 1 Laboratory of Clinical immunopathology, Federal State Budgetary Scientific Institution “Research Institute of Fundamental and Clinical Immunology” (RIFCI), 630099 Novosibirsk, Russia; 2 Department of Medicine, Novosibirsk State University, 630090 Novosibirsk, Russia; 3 Laboratory of Cluster and Supramolecular Chemistry, Nicolaev Institute of Inorganic Chemistry, 630090 Novosibirsk, Russia

**Keywords:** nanoparticles, cucurbiturils, reactive oxygen species, immunocompetent cells, RBC, platelets, tumor cells

## Abstract

Reactive oxygen species (ROS) are highly reactive chemical molecules containing oxygen. ROS play an important role in signaling and cell homeostasis at low and moderate concentrations. ROS could be a cause of damage to proteins, nucleic acids, lipids, membranes and organelles at high concentrations. There are a lot of cells that can produce ROS to maintain functional activity. It is known that metal nanoparticles can increase production of ROS in cells. However, the effect of cucurbiturils on ROS production is still unknown. In our study, we evaluated production of ROS by the immune (T-, B-lymphocytes, NK-cells) and non-immune cells (red blood cells, platelets), as well as tumor cells line (1301, K562) after treatment with cucurbiturils in vitro. Assessment of reactive oxide species (ROS) were provided by using dihydrorhodamine 123 (DHR 123). Fluorescence intensity and percentage DHR123 were measured by flow cytometry. Platelets, erythrocytes and activated T-helpers were changed the level of ROS production in response to stimulation with cucurbiturils. It was found that the percentage of these ROS-producing cells was reduced by cucurbiturils. Thus, cucurbiturils may affect the production of ROS by cells, but further research is needed in this area.

## 1. Introduction

The 21st century is notable for the development of nanomedicine. The nanomaterials economics is estimated at billions of dollars. The most prospective and rapidly developing area is nanoscale drug delivery systems. Various biocompatible nanomaterials can be used as drug delivery systems. Polymeric nanomaterials are of great interest to biomedical and pharmacotherapeutic applications such as cucurbiturils, dendrimers, glycyrrhizic acid, etc.

Cucurbiturils (CB[n]) are supramolecular cavitands made of a condensation reaction of urea, glyoxal and formaldehyde [1]. CB[n] are polymeric organic agents, consisting of the monomeric structural unit of glycoluril. The amount of glycoluril determines the number of the homologue, so the more glycoluril fragments are the larger the cavity of each following cucurbituril is, while their height is constant—9.1 Å [2]. CB[n] form guest–host inclusion complexes with cationic and neutral molecules. CB[n] is used as a drug delivery system to enhance therapeutic effect. Complexation CB[n] with various medicinal substances allows to minimize the side effects of the medicine, increase their solubility, apply a lower concentration and a prolonged effect of drugs [3].

Despite advantages of cucurbituril, the issue of biocompatibility remains open, as well as the mechanisms of toxicity of cucurbituril. It is especially significant to research cucurbiturils by way of their clinical application. Their interaction with biological systems requires an approximate regard, as they might have a toxic effect on the cells of the organism. It is known that CB[n] were exhibited cardio- and myotoxicity [4]. The interactions with the immune system have not been well studied. The interaction of CB[n] with immunocompetent cells are challenges to be faced before achieving the practical aim of clinical applications, because the immune system is complex, multifactorial, multidimensional and self-regulating system. It is important to conceive that immunotoxicology is the greatest part in the research of any xenobiotic drugs, including nanoscale ones.

There are three mechanisms of the toxicity of nanomaterials: oxidative stress through reactive oxygen species (ROS) generation, direct cell damage and genotoxicity [5]. Previously we have assumed that the toxicity of cucurbiturils is not associated with a direct damaging effect on cells [6]. Indeed, today, it is known that CB[n] can across the cell membrane [7], which means that, theoretically, they may be interacted with any elements inside the cell, including cellular organelles such as mitochondria. Thus, an important step in the study of cucurbiturils is the assessment of the production of total reactive oxygen species in the cells of the organism.

Reactive oxygen species are permanently generated by the cells of the organism as a product of oxygen metabolism. ROS include superoxide anion radical, singlet oxygen, hydroxyl radical and hydrogen peroxide. ROS are a double-edged sword: it maintains a highly reactive oxygen atom, freely interacting with other molecules in the cell, and could lead to damage to DNA, RNA, proteins, lipids, or to cell death, but ROS also take part in various biological functions, including survival, growth, proliferation, cell differentiation and signaling pathways of immune system. ROS are associated with a wide range of pathological processes such as chronic inflammation, tumors and age-related diseases [8]. We have focused on various effects of ROS depending on the concentration of ROS in cells. However, the effect may be associated with a specific form of oxygen (H_2_O_2_, O_2_^−^, etc.). Conditionally, it is possible to distinguish low, medium and high concentrations of ROS. Nowadays there are no reference index of concentration ROS in each population of cell of each donor or patient. It is not clear what is considered low or high dosages. However, the participation of ROS in physiological and pathological processes in various cells is unquestionable. Different studies have clearly defined the effect and concentration of ROS to compared with ‘normal values’, with proper, adequately functioning physiology of the cell. For example, macrophages permanently produce reactive oxygen species (ROS) to realize their intracellular functions (signaling, differentiation, gene expression) and to release in response to phagocytosis or stimulation by various chemical agents [9]. Reactive oxygen species, especially H_2_O_2_, which diffuses through various cellular compartments could be a secondary messenger in cellular signaling. Phagocytic cells can produce large amounts of H_2_O_2_ to activate dendritic cells (DC) in the subsequent initiation of an antigen-specific immune response [10]. ROS produced by macrophages also have immunosuppressive properties and induce Treg cells [11]. Moreover, the effects of ROS would be dependent on the exposition, location [12,13,14]. There is a lot of data regarding the production of reactive oxygen species by phagocytes, so our study focused on nonclassical ROS-producing cells. For example, NK cells require reactive oxygen species for the lysis of pathogens [15,16]. T cells also produce reactive oxygen species during oxidative metabolism, which take part in regulating T cell functions [17]. T cells with low ROS levels demonstrated impaired production of interleukin-2 and antigen-specific proliferation [18]. In other side metabolic rearrangement (during T-lymphocyte activation) affecting ROS production: high ROS levels are involved in the induced activation of T-cell death [19,20], moderate ROS levels are required for the function of the perforin-granzyme complex [21,22]. ROS are essential for activated B cells to enhance signal transduction through the B cell receptor. The conventional levels (maybe low or moderate dosages) of ROS are necessary to perform B-cells functions; for example, switching the dormant state of cells and recirculation to activation, proliferation, mass production of antibodies and survival [23]. High ROS levels (to compare with low and moderate values) prevents these cells from producing immunoglobulins [24]. In other words, the local accumulation of ROS is the increase in their concentration to high values.

ROS are also constantly produced by erythrocytes through oxygen transport, establishing a high level of oxidative stress, which significantly reduces their lifespan [25]. In response to an infectious agent or exogenous substance, ROS production by erythrocytes sharply increases [26,27]. ROS are generated by activated platelets and play an important role in the regulation of platelet responses to collagen formation and collagen-mediated thrombus formation [28]. ROS production in a platelet population can be significantly increased by either TRAP (a potent agonist that induces granule release) or PMA (a classical inducer of ROS generation) [29]. ROS are known to play a key role in intra-platelet signaling and subsequent platelet activation [30]. Perhaps the same concentration of ROS will be high for a B-lymphocyte or platelets, but moderate for an erythrocyte or macrophage.

Today there is a great variety of nanoparticles, so their effects on the generation of ROS in different living cells are inequitable. For example, superparamagnetic iron oxide nanoparticles generate high ROS levels by PC 12 cell lines and induces cell apoptosis [31]. Treatment of fibroblasts by long horizontal nanowires in vitro caused cell division to cease, due to the high levels of ROS damage to DNA; while vertical nanowires induce cell motility and increase the proliferation rate [32]. Sohaebuddin et al. investigated the effect of the toxicity of chemical composition of nano-TiO_2_, nano-SiO_2_, and multi-walled CNTs to 3T3 fibroblasts, RAW 264.7 macrophages and telomerase-immortalized (hT) bronchiolar epithelial cells [33]. 

The influence of more complex nanomaterials on cells and ROS production is not so clear. For example, among fullerenes, C60, which has a spherical shape, there are water-soluble, monodisperse or colloidal fullerene aggregates. They induce superoxide anions and lipid peroxidation, which lead to cytotoxicity. Various functional groups attached to the surface of fullerenes are critical determinants of fullerene-induced toxicity. Modification of the fullerene surface by the addition of one or several malonyl groups [34], carnosine [35] are made fullerene derivatives with antioxidant activity. Thus, fullerenes with structural and surface modifications have many potential applications based on their capabilities in the implementation of prooxidant (generation of free radicals) or antioxidant (absorption of free radicals) activities. The structure of cucurbiturils is similar to fullerenes.

There are a few data regarding the effect of nanomaterial without modifications or complexation with other substances on ROS production by cells. The data of the effect of cucurbiturils on ROS generation were not found in the available literature. However, cucurbit [8]uril was used as a supramolecular nanocapsule. It was designed by the complexation of azobenzene (Azo) and methylviologen (MV) with cucurbit[8]uril. This supramolecular nanocapsule with activities of both glutathione peroxidase and superoxide dismutase could simulate the defense system against intracellular reactive oxygen species [36]. However, the influence of cucurbiturils on ROS generation has not been studied. The level of ROS generation using the designed nanomaterials depends on the chemical nature of the nanoparticles [37]. In summary, there are critical determinants that can affect the production of ROS by nanomaterials. These critical determinants include: size, shape, surface of particles, surface charges, surface containing groups, dissolution of particles, aggregation, mode of interaction with cells, inflammation and pH of the environment. The larger the contact area of the nanoparticles, the higher their biocompatibility. The critical determinants can afford the nanoparticles significant differences in interactions with biological systems, which requires research in this area.

Side effects may be associated with the production of reactive oxygen species by cells during treatment with some agents. Therefore, it is important to take into account any effect of nano-agents on cells when it injects to the organism. Due to the fact that ROS play an important role in the function and death of cells, we decided to evaluate ROS production in cells by treatment of cucurbiturils.

## 2. Results

**Figure 1:**
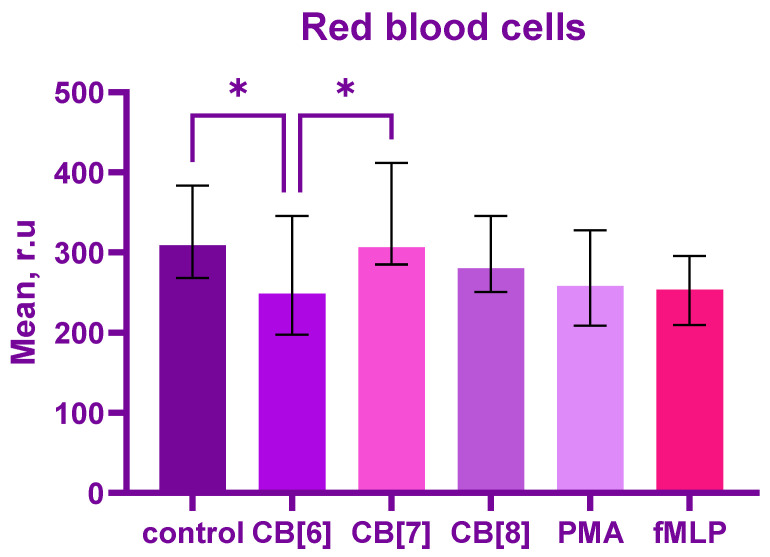
Graph of the fluorescence intensity of erythrocytes, producing reactive oxygen species in response to stimulation with cucurbiturils (CB[6,7,8]) during 1 h. Data are presented as median ± interquartile range with n = 10. * *p* < 0.05 by employing one-way ANOVA, Friedman test.

CB[6] significantly reduced the production of reactive oxygen species in erythrocytes compared to control and CB[7], *p* = 0.02. No differences were found between CB[6] and CB[8]; CB[7] and CB[8]; control and positive control; and cucurbiturils and positive control (Figure 1).

**Figure 2:**
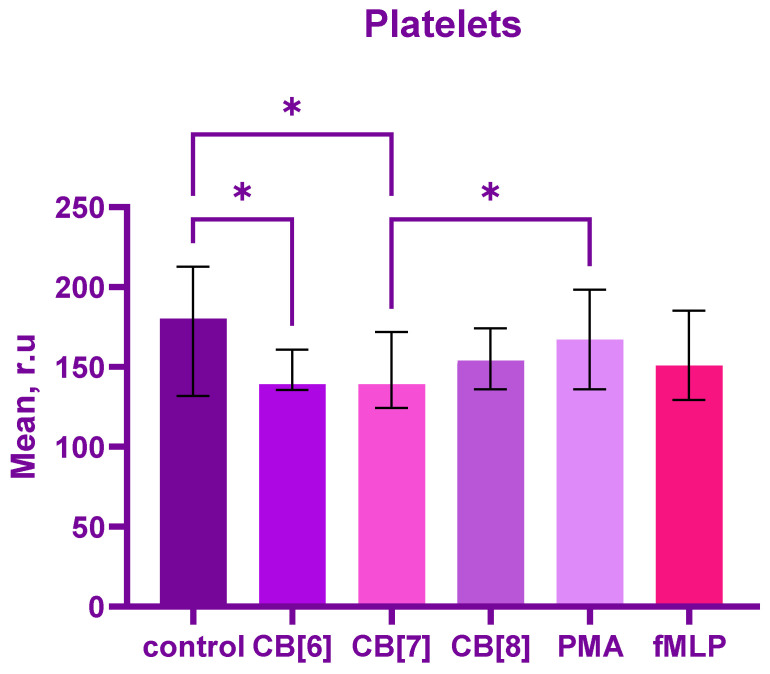
Graph of the fluorescence intensity of platelets, producing reactive oxygen species in response to stimulation with cucurbiturils (CB [6,7,8]) during 1 h. Data are presented as median ± interquartile range with n = 10. * *p* < 0.05 by employing one-way ANOVA, Friedman test.

Moreover, CB[6] and CB[7] reduced the production of reactive oxygen species in platelets compared to the control (*p* = 0.04 and *p* = 0.01, respectively). Interestingly, CB[7] reduced ROS production compared to PMA positive control, *p* = 0.01 and CB[6] had a tendency compared to PMA, *p* = 0.05 (Figure 2). No significant difference was found between cucurbiturils, CB[8] and control.

**Figure 3:**
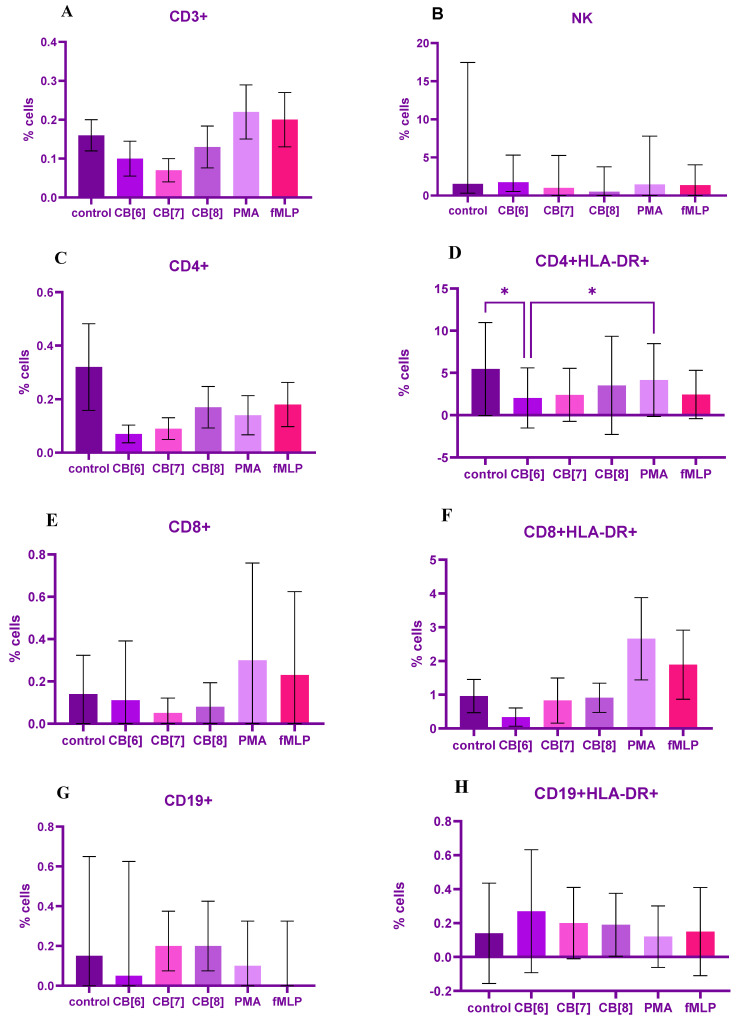
Graph of the percentage of cells of various subpopulations of lymphocytes, producing reactive oxygen species (**A**–**H**) in response to 24-h stimulation with cucurbiturils (CB [6,7,8]). Data are presented as median ± interquartile range with n = 10. * *p* < 0.05 for comparing CB[6] group with control and PMA by employing one-way ANOVA, Friedman test.

Interestingly, CB[n] had low effect on mononuclear cells. Percentage of ROS-producing cells among specific population was great varied among donors. Thus, CB[6,7,8] had no influence on the production of reactive oxygen species by CD3+ population cells (Figure 3A). However, CB[6] and CB[7] were reduced the percentage of ROS-producing cells among the CD4+ population compared to fMLP (*p* = 0.05 and *p* = 0.07, respectively) and CB[7,8] were reduced the percentage of ROS-producing cells compared with control (*p* = 0.08) (Figure 3C).

The percentage of ROS-producing cells among the culture of activated CD4+HLA-DR+ cells was significantly reduced compared with control and PMA (*p* = 0.01 and *p* = 0.02, respectively) under the influence of CB[6]; but also CB[7,8] were insignificant decreased the percentage of ROS-producing cells among these cells compared with control and PMA (*p* = 0.08 for both CB[n] for control, *p* = 0.09 for both CB[n] for PMA), and no significant difference was found between high, low stimulants and control (Figure 3D).

At the same time, the percentage of ROS-producing cells among the population of CD8+ cytotoxic T-lymphocytes decreased due the influence of CB[6,7] (*p* = 0.05) (Figure 3E), although only CB[6] was effected on produce of ROS by activated CD8 + HLA-DR+ compared with PMA (*p* = 0.06). No difference was found between control and stimulated controls (Figure 3F).

Graphs of B-lymphocytes showed an increase ROS production in response to the effect of CB[7], CB[8] compared with fMLP (*p* = 0.09 and *p* = 0.07, respectively) (Figure 3G). Additionally, activated B-lymphocytes also increased the level of ROS compared with the control (*p* = 0.08) under influence of CB[6] (Figure 3H) and no significant differences were found between the control and the low stimulant in both these population of cells.

There were no significant differences in ROS production by NK-cells compared to controls (Figure 3B).

It is important that we reported a trend, but not a significant difference between groups, where *p* ≥ 0.05.

**Figure 4:**
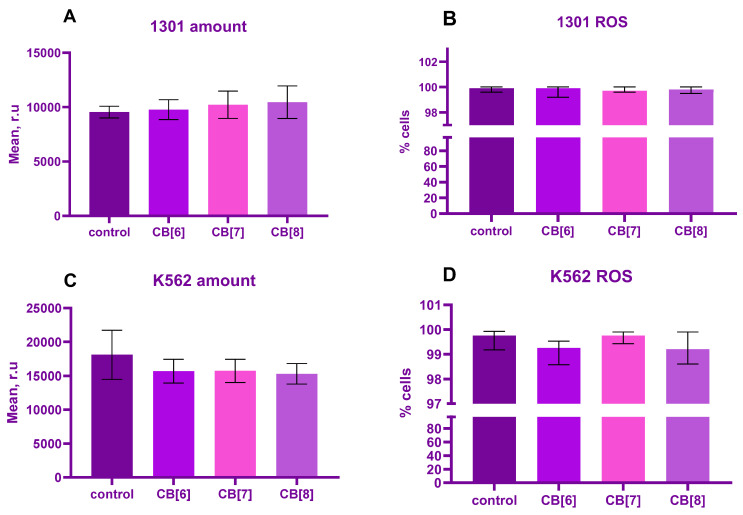
Graph of the amount of 1301 tumor cells (**A**) and the percentage of cells producing reactive oxygen species (**B**), Graph of the amount K562 tumor cells (**C**) and the percentage of cells producing reactive oxygen species (**D**) in response to 24-h stimulation with cucurbiturils (CB[6,7,8]). Data are median ± interquartile range (n = 6), (Kruskal–Wallis one-way analysis of variance). Notes: (**A**,**C**)—fluorescence intensity; (**B**,**D**)—number of ROS-producing cells among this cell’s population.

Unfortunately, we found no significant differences between control and cucurbiturils in ROS production by tumor cell lines 1301 and K562 (Figure 4). However, it is known that other polymeric nanoparticles such as dendrimers can cause changes ROS levels in the 1301 cells and liposomes in the K562 tumor cells [38,39].

## 3. Discussion

It is known that cucurbiturils at low concentrations no have a cytotoxic effect on organism cells. However, it is necessary to take into account the probability of ROS generation by organism cells due the influence of cucurbiturils. There is no reference to the effect of cucurbiturils on the ROS levels in the cells such as platelets, erythrocytes and immunocompetent cells.

In our study, we obtained interesting results regarding the production of ROS by erythrocytes and platelets, immunocompetent cells after treatment by cucurbiturils. Cucurbiturils decreased ROS production by these cells, probably, which is associated to the fact that cucurbiturils may activate the antioxidant defense system or they have ability to antioxidant activity. It is known that cucurbiturils, when complexed with antioxidant substances, can increase their antioxidant activity [40,41,42].

Different ROS levels into tumor lines can lead to their death or malignancy due effecting of the various molecules [43,44,45]. These molecules either increase or decrease ROS levels; therefore, it was necessary to research effect of cucurbiturils on ROS production by different tumor cells.

Interestingly, the analogue of cucurbiturils (molecules forming complexes according to the guest-host type)–cyclodextrin and calixarenes have not cytotoxic activity against the K562 tumor line as also cucurbituril. However, complex formation or modification of the nanosubstance’s structure increases their antitumor activity in regard chronic myeloid leukemia [46,47]. Increased antitumor activity was associated with apoptosis of these cells, but it is not clear whether this is due to the generation of reactive oxygen species. It is known that a signaling pathway induces ROS-mediated apoptosis [48,49]. At the same time there are data regarding the interrelation between ROS generation and apoptosis in the K562 tumor line, but the inducer of apoptosis directly played an important role in this signaling pathway. For example, inorganic nanoparticles were capable of inducing apoptosis in human K562 tumor cell line through generation of reactive oxygen species [50], while docetaxel induced ROS-independent apoptosis [51].

In our study cucurbiturils had no effect to production ROS levels by these leukemia tumor lines (1301, K562), which may be associated with internalization into the cell; nowadays, it is not known that cucurbiturils are able to internalize into these cells. However, the complex formation of cucurbiturils with platinum increases the antitumor toxicity in K562 cells. Research should be conducted into the ROS levels producing by tumor cells in vitro with treatment by the complex of cucurbit[7]urils and platinum compounds to investigate whether the antitumor effect is associated with the generation of ROS.

Moreover, in our study, we received a lot of data that had a trend. It is possible that the lack of significant difference is associated with a small group, and if the number of donors was increased, then there may be *p* value of less than 0.05.

An important part of the discussion is the pathophysiological manifestations of the effect of CB-induced ROS variation on cell biological functions such as proliferation, apoptosis and hemolysis. We previously reported on the effect of cucurbit[n]urils on these processes [6,52], so we can make some assumption.

It is known that an increase in the concentration of ROS can lead to ROS-induced hemolysis [53]. RBC are involved in maintaining the redox balance caused to the permanent generation of ROS due to the presence of a hemoglobin molecule. Erythrocytes in specific conditions can cause oxidative stress associated with hyperproduction of ROS and oxidative stress is associated with many diseases such as chronic kidney and cardiovascular diseases [54]. Some studies have reported that antioxidant interventions in various situations have proved beneficial to erythrocytes [55]. For example, antioxidants protect RBC from damage and do not reduce their activity. Similarly, oxidant treatment has been shown to affect erythrocyte deformability ex vivo and demonstrated that high ROS levels to diminish ability of erythrocyte to deformability [26]. In our previous studies, CB[7] caused hemolysis of erythrocytes; however, we had no significant differences in the effect of CB[7] on ROS production. At the same time, CB[6] had no effect on hemolysis, but CB[6] had ability to reduce the concentration of ROS. Accordingly, cucurbiturils lead to lack of oxidative stress, and cucurbiturils are not able to worsen or to be the cause of some ROS-related diseases.

ROS play an important role in platelet activation. High levels of ROS hyperactivate platelets, their adhesion, leading to a procoagulant platelet phenotype and apoptosis [56]. Cucurbiturils, in particular CB[6] reduced ROS levels in platelets, thus minimizing the risk of thrombosis in diseases associated with oxidative stress.

Low concentrations of ROS in T cells are a necessary condition for cell survival, and increased high ROS-levels can lead to apoptosis/necrosis [57]. A decrease in the proliferative activity and an increase in apoptotic cells among CD4+ T-lymphocytes and even an increase in expression of HLA-DR on these cells in response to CB[6] is not associated with generation of reactive oxygen species, since we have shown that CB[6] reduces the level of ROS. Interestingly, the expression of HLA-DR on dendritic cells is enhanced in response to increased concentrations of ROS [58]. This means that cucurbiturils, in particular CB[6], exhibit their immunomodulatory and cytostatic effects not associated with ROS generation.

In summary, it is necessary to note the fact that in the studies of various authors’ groups, those cell lines that are arrange to the production of high ROS levels could be used. Therefore, in our study, we focused on non-classical cells, which have the ability to production of various ROS levels, possibly including low, moderate and high concentrations.

## 4. Materials and Methods

### 4.1. Materials, Reagents and Preparation

Cucurbit[n]urils (n = 6, 7 and 8) were synthesized, purified and kindly provided by the Nikolaev Institute of Inorganic Chemistry (Novosibirsk, Russia). Before the experiments, CB[6] and CB[7] were prepared at a concentration 0.3 mM; CB[8] at 0.01 mM in the culture medium RPMI-1640 (Paneco, Russia). Reagents: gentamicin (Dalfarma, Khabarovsk, Russia), tienam (Merck Sharp & Dohme Corp., Kenilworth, NJ, USA), FCS (Hyclone, Chicago, IL, USA), Ficoll (BioClot, Aidenbach, Germany), urografin 76% (Schering, Berlin, Germany), Human Platelet Isolation Kit (BioVision, CA, USA), tabs for preparing phosphate-buffered saline (PBS) (AppliChem GmbH, Darmstadt, Germany), Na2EDTA (Helicon, Moscow, Russia), N-formyl-methionyl-leucyl-phenylalanine (fMLP) (Celonic Deutschland GmbH & Co. KG, Heidelberg, Germany), Phorbol-12-myristate 13-acetate (PMA) (Celonic Deutschland GmbH & Co. KG, Heidelberg, Germany), Dihydrorhodamine 123 (DHR) (Celonic Deutschland GmbH & Co. KG, Germany), Wash Buffer (Celonic Deutschland GmbH & Co. KG, Heidelberg, Germany), ProClinR 300 (Celonic Deutschland GmbH & Co. KG, Heidelberg, Germany), CD3 PerCP-Cy5.5 (BioLegend, CA, USA), CD4 PE-Cy7 (BioLegend, CA, USA), CD14 PE Cy7 (BioLegend, CA, USA), CD16 APC (BioLegend, CA, USA), CD19 APC Cy7 (BioLegend, CA, USA), CD56 APC Cy7 (BioLegend, CA, USA), HLA-DR APC (BioLegend, CA, USA), CD45 PE-Cy7 (BioLegend, CA, USA).

Human T-cell leukemia cell line 1301 and human chronic myelogenous leukemia cell line K562 (European collection of authenticated cell cultures, Sigma Aldrich, Merck KGaA, Darmstadt, Germany) were used. 

The Ethical Committee of RIFCI, Russia, approved the study design and the recruitment of subjects. Subjects provided written informed consent. The relevant guidelines and regulations were followed when performing the experiments.

### 4.2. Isolation and Cultivation of Cells

#### 4.2.1. Isolation and Cultivation of PBMC

Peripheral blood was obtained from 10 volunteers (average age 31.5 ± 8.7 years). Peripheral blood mononuclear cells (PBMCs) were isolated by density-gradient on a Ficol-urografin (1.078). The PBMCs were washed twice with phosphate-buffered saline and EDTA by centrifugation at 300× *g* for 5 min. The cells were counted in 3% acetic acid using a Goryaev chamber. Isolated PBMCs at a concentration of 1 × 10^5^ cells/well were cultivated in a culture medium RPMI-1640, supplemented with 50 mg/mL gentamicin, 25 mg/mL tienam and 10% FCS in a 96-well plate (TPP, Trasadingen, Switzerland) for 24 h in 37 °C and a humidified atmosphere of 5% CO_2_.

#### 4.2.2. Isolation and Cultivation of RBC

A group of volunteers (n = 10, average age 31.9 ± 8.6 years) was formed to determine intracellular ROS by CB[6,7,8]. The separation of erythrocytes from plasma was performed by 1400 g centrifugation with ficoll (which provides the density gradient) for 25 min. The erythrocytes were washed twice with PBS by centrifugation 800× *g* for 10 min after plasma removal. Then the erythrocytes were suspended in PBS to count in a Goryaev chamber using the standard formula: N = m × 4000 × s/q, where “N” is the number of erythrocytes per 1 mm^3^ of blood; “m” is the number of erythrocytes in a certain number of small squares; «s” is dilution of blood; “q”—the number of small squares counted in chamber. The erythrocytes were used immediately.

Next, isolated RBCs at a concentration of 1 × 10^5^ cells/well were cultivated in a culture medium RPMI-1640, supplemented with 50 mg/mL gentamicin, 25 mg/mL tienam in a 96-well plate for 1 h in 37 °C and a humidified atmosphere of 5% CO_2_.

#### 4.2.3. Isolation and Cultivation of Platelets

For human platelets, venous blood was collected into a tube anti-coagulated with 3.8% natrium citrate. Platelets were isolated from volunteers (n = 10, average age 33.3 ± 9.0 years) using the Human Platelet Isolation Kit. Blood donors did not taken aspirin or antiplatelet medications or agents for at least 48 h. Storage conditions, reagent preparation, platelet isolation and viability assay were performed according to the manufacturer’s protocol. Platelets were counted in PBS in the Goryaev chamber according to the principle:(Platelets counted in 5 squares) × (Dilution factor)/Area × Depth = (Platelets) × 100. Platelet viability was measured using a commercial kit (Human Isolation Kit, Biovision, CA, USA) according to the manufacturer’s protocol. The total number of platelets was determined in bright-field by counting the five hemocytometer’s large squares. The cells stained with vital dye were counted using fluorescence microscopy in the same way. The percentage of living cells was determined by the formula: number of stained platelets/total number of platelets × 100%. Microscopy was performed with the “Axioskop-40” fluorescence microscope (ZEISS, Jena, Germany) equipped with a high-pressure mercury lamp HBO 50 W, with Zeiss interference filter sets (Set № 13) and CCD-chamber «AxioCam 503 mono» (at 1936 × 1460 px resolution and 14-bit capacity). Platelets images were captured with software package «ZEN-2012» (ZEISS, Germany). An exposure time was settled automatically for each image. We used the same condition of cultivation for platelets as for RBC.

#### 4.2.4. Cultivation of Tumor Lines

The K562 and 1301 suspension tumor cell lines obtained from the manufacturer were cultured according to standard conditions with Culture Medium RPMI-1640 + 2 mM Glutamine + 10% Fetal Bovine Serum (FBS). Cultures were maintained between 100,000 to 1,000,000 cells/mL; 5% CO_2_; 37 °C. To assess ROS levels, cells at a concentration of 1 × 10^5^ cells/well were cultivated in a 96-well plate. The results are the mean of three experiments in duplets.

### 4.3. Treatment of Cells by Cucurbit[n]urils (n = 6, 7, 8)

For assay, PBMCs and cells of tumor lines K562, 1301 were treated with CB[6] and CB[7] at concentrations of 0.3 mM (IC50~0.5 mM as reported by Uzunova VD et al. [59]) and CB[8] at 0.01 due low solubility range in relevant culture medium during 24 h (5% CO_2_; 37 °C). On the day of the experiment, the tumor cells were incubated without PMA and fMLP stimulation. RBCs and an isolated live platelets were treated with CB[6], CB[7] at concentrations of 0.3 mM and CB[8] at 0.01 mM during 1 h (5% CO_2_; 37 °C).

After incubation with cucurbit[n]urils (n = 6, 7, 8) the samples were washed and collected in tubes for further preparation (par. 2.4).

### 4.4. Measurement of Intracellular Reactive Oxygen

The level of intracellular ROS was assessed using the fluorescent dye DHR-123 (PE). After treatment with cucurbiturils, cells were stimulated with 1 mMol fMLP solution (as a poor physiological stimulant—“low control”) and 1.62 mMol PMA solution (as a strong stimulant—“high control”) and wash buffer (as a “negative control”) for 10 min in a water bath at 37 °C (physiological values). Next, the cells were resuspended and DHR-123 was added to cells by mixing thoroughly. Samples were incubated for 10 min at 37 °C in a closed water bath. Then, samples were washed with 3 mL Wash Buffer by centrifugation 0.3× *g* for 5 min at 2–8 °C from excess dye (DHR-123). Finally, the fluorescence signal was recorded by flow cytometry using a FACSCanto II cytometer (BD, Franklin Lakes, NJ, USA) with software Diva 6.0 (BD).

### 4.5. Phenotype of Cells

We determined the phenotypes of T-cells as CD3+CD4+ for T-helpers and CD3+CD4- for T-killer cells; B cells as CD3-CD19+; NK cells as CD3-CD16+CD56+, monocytes as CD3-CD14+ CD16+ and activated cells by HLA-DR marker. Expression of these molecules was performed by staining cells with fluorochrome-conjugated monoclonal antibodies specific for cluster differentiation. Samples were analyzed on a FACS Canto II flow cytometer with standard set threshold on forward scatter (FSC) for PBMCs. Production of ROS was analyzed from the gates CD3+CD4+, CD3+CD4-, CD3-CD19+, CD3-CD16+CD56+, CD3-CD14+CD16+.

For platelets and erythrocytes, the threshold was set to a low value for forward scattering (FSC) compared to PBMCs.

### 4.6. Statistical Data Analysis

All the data were evaluated statistically by using the GraphPad Prism software (Version 9.0.0 for Windows; GraphPad Software Inc., San Diego, CA, USA). Kruskal–Wallis one-way analysis of variance and Friedman test were used to assess the differences between groups and a value of *p* < 0.05 was considered statistically significant. The data were expressed as median ± interquartile range.

## 5. Conclusions

The toxicity of nanosystems and nanoparticles is associated with increased generation of reactive oxygen species. However, we have demonstrated that the cucurbiturils is also capable of reducing ROS production; accordingly, there are other ways of nanotoxicity. Our results showed that not only different nanoparticles, but also different cells can react to the contrary. Activated cells, platelets and red blood cells are more able to change ROS levels due influence of cucurbiturils. The mechanisms of ROS’ nanotoxicity need to be investigated in detail, for example to increase the incubation time with nanoparticles and to use different cell types to establish sensitivity to any particular nanoparticles, to measure mitochondrial transmembrane potential, to assess ROS defense machinery. These studies are basic for the further successful application of the nanotechnology strategy in the treatment of cancer or other diseases.

## Data Availability

Not applicable.
